# Drug-Coated Balloon After Intravascular Lithotripsy: Procedural and Clinical Outcome of the “Crack and Drug” Strategy

**DOI:** 10.1016/j.jacadv.2025.102250

**Published:** 2025-10-24

**Authors:** Federico Oliveri, Martijn J.H. van Oort, Akshay A.S. Phagu, Ibtihal Al Amri, Brian O. Bingen, Valeria Paradies, Gianluca Mincione, Bimmer E.P.M. Claessen, Aukelien C. Dimitriu-Leen, Joelle Kefer, Hany Girgis, Tessel Vossenberg, Alessandro Mandurino-Mirizzi, Frank Van der Kley, J.Wouter Jukema, José Montero-Cabezas

**Affiliations:** a Department of Cardiology, Leiden University Medical Center, Leiden, the Netherlands; b Department of Cardiology, Maasstad Ziekenhuis, Rotterdam, the Netherlands; c Department of Cardiovascular Medicine, Humanitas Research Hospital-IRCCS, Rozzano, Italy; d Department of Cardiology, Amsterdam University Medical Center, Amsterdam, the Netherlands; e Department of Cardiology, Radboud University Medical Center, Nijmegen, the Netherlands; f Department of Cardiology, Saint-Luc Bruxelles, Belgium; g Department of Cardiology, Jeroen Bosch Ziekenhuis, Den-Bosch, the Netherlands; h Department of Cardiology, Frisius Medisch Centrum, Harlingen, the Netherlands; i Division of Cardiology, Vito Fazzi Hospital, Lecce, Italy; j Department of Experimental Medicine (DiMeS), University of Salento, Lecce, Italy; k Netherlands Heart Institute, Utrecht, the Netherlands

**Keywords:** coronary calcification and DCB, DCB, intracoronary lithotripsy, IVL and DCB

## Abstract

**Background:**

Drug-eluting stents (DESs) after intravascular lithotripsy (IVL) have proven effective in calcified coronary lesions. However, evidence supporting drug-coated balloons (DCBs) after IVL remains limited.

**Objectives:**

We aimed to compare the technical success and 1-year clinical outcomes of IVL + DES vs IVL + DCB strategies for the treatment of balloon-crossable calcified coronary lesions.

**Methods:**

Patients undergoing percutaneous coronary intervention with IVL from the prospective BENELUX-IVL registry were included. Participants were stratified into DCB or DES groups based on post-IVL treatment strategy. The primary endpoint was procedural success, a composite endpoint defined as successful IVL catheter crossing with residual stenosis <30%, final TIMI flow grade 3, and no in-hospital major adverse cardiac events (MACE). The primary safety endpoint was in-hospital MACE, including cardiac death, nonfatal myocardial infarction, or target vessel revascularization.

**Results:**

Among 579 patients, 45 (7.8%) were treated with DCB after IVL. Baseline characteristics, clinical presentation, and SYNTAX scores were comparable between groups. Chronic total occlusions (17.8% vs 6.8%; *P* < 0.01) and in-stent restenosis lesions (60.0% vs 29.6%; *P* < 0.01) were more common in the DCB group. Intraprocedural complications were low, with no significant difference (8.9% vs 5.8%; *P* = 0.41) between DES or DCB strategies. No bailout DES implantation was required in the DCB arm. Procedural success (89.4% vs 91.7%; *P* = 0.55) and in-hospital MACE rates (0% vs 2.1%; *P* = 0.33) were comparable among the 2 strategies.

**Conclusions:**

In calcified coronary lesions, DCB after IVL demonstrates excellent efficacy and safety, achieving high technical success with low device-dependent adverse event rates.

Over the past decades, percutaneous coronary interventions (PCIs) have undergone a remarkable evolution. The transition from balloon angioplasty (POBA) to drug-eluting stents (DESs) has significantly enhanced procedural and clinical outcomes, markedly reducing restenosis and the need for target lesion revascularization.^1^^,^^2^ More recently, drug-coated balloons (DCBs) have emerged as a promising alternative, aiming to combine the advantages of a stent-free approach, reminiscent of POBA, with the proven efficacy of DES.^3^, ^4^, ^5^ Although DCB has proven effective in several clinical settings—including in-stent restenosis (ISR), small (≤2.75 mm) and even in larger coronary vessels—its role in treating calcified coronary lesions remains less established.^3^, ^4^, ^5^ DCB may offer unique benefits in complex anatomical settings, such as calcified bifurcations, trifurcations, or chronic total occlusions (CTOs).^6^ However, several challenges must be addressed. One key limitation is the lack of radial force, which makes DCBs more susceptible to elastic recoil, particularly in highly calcified lesions. In addition, calcium plaques may interfere with drug diffusion, potentially impairing DCB pharmacokinetics and therefore reducing efficacy.^7^ These limitations can, however, be addressed through calcium modification techniques.^8^ In particular, intravascular lithotripsy (IVL) has demonstrated excellent results in balloon-crossable calcified lesions by creating controlled fractures within calcium plaques, thereby improving coronary compliance and potentially improving drug pharmacokinetics.^9^, ^10^, ^11^, ^12^ Despite the increasing adoption of IVL, the optimal post-IVL treatment strategy is quickly evolving. Although DES provides structural support and sustained drug delivery, DCB preserves native vessel integrity while still delivering an antiproliferative agent. However, whether DCB can achieve similar outcomes to DES after IVL remains unknown. Thus, the aim of this study is to compare the procedural and clinical outcomes of IVL patients treated with either DCB or DES.

## Methods

### Population and data collection

The BENELUX-IVL registry is an international, multicenter, prospective study (NCT06577038) involving all-comer patients aged ≥18 years who underwent IVL during PCI. From the whole registry, participants were stratified into DCB or DES groups based on post-IVL treatment strategy. For all the IVL procedures, the Shockwave Intravascular Lithotripsy Coronary System (Shockwave Medical) was used. Procedural technical decisions—including timing, balloon size, number of pulses, maximum pressure, as well as the use of high-pressure predilation and postdilation, other debulking devices, and intracoronary imaging—were made at the discretion of the operating physician and systematically recorded in procedural documentation. We collected demographic, procedural, clinical, and follow-up data from the hospital's electronic health records. Patients who could not provide informed consent were excluded from the study. Angiographic and imaging data were analyzed in a centralized core laboratory. The examination of clinically gathered data received approval from the local ethics committees of each participating institution.

### Definitions and imaging analysis

In accordance with the most recent European Association of Percutaneous Cardiovascular Interventions consensus, we scored coronary calcification severity using intravascular ultrasound (IVUS) parameters (360-degree calcification, calcium extending over 270-degrees and measuring ≥5 mm in length, detection of a calcified nodule, or a vessel diameter <3.5 mm) or optical coherence tomography (OCT) score (2 points for a maximum angle >180°, 1 point for a maximum thickness >0.5 mm, and 1 point for a length >5 mm).^13^, ^14^, ^15^ If intravascular imaging was not available, radiopaque angiographically densities visible without cardiac motion before contrast injection, affecting both sides of the coronary artery wall, were considered a hallmark of severe calcification.^13^ As an alternative, the evidence of “dog-bone effect” (underexpansion of the central portion of a 1:1 noncompliant balloon inflated at high atmospheres) was considered an indication for IVL use. Quantitative coronary analysis (QCA) and intracoronary imaging were retrospectively assessed offline for vessel and stent characteristics. QCA was carried out pre-IVL and post-IVL, using Medis Suite QCA (2D/3D) software (Medis Suite 4.0.24.4, Medis Medical Imaging System BV). Measurements included minimum lumen diameter, minimum lumen area (MLA), reference vessel diameter, percent area stenosis and acute gain. Analysis of IVUS and OCT were performed using QCU-CMS 4.69 (Leiden University Medical Center). Intracoronary imaging parameters were evaluated based on the European Association of Percutaneous Cardiovascular Interventions consensus on the clinical use of intracoronary imaging.^16^ Intracoronary imaging measurements included the reference vessel diameter, area stenosis, pre-PCI MLA, max Ca^2+^ angle, post-PCI minimum stent area (MSA). The eccentricity index was calculated by subtracting the minimum lesion diameter from the maximum lesion diameter at the MLA or MSA, and then dividing this difference by the maximum lesion diameter. Stent expansion at the MSA was assessed by dividing the MSA by the reference vessel area.

### Study endpoints

The primary endpoint was procedural success, defined as the successful delivery of the IVL catheter across the target lesion and delivery of pulses and residual target lesion <30% (assessed by QCA), TIMI flow grade 3 and no in-hospital major adverse cardiovascular events (MACE). The primary safety endpoint was in-hospital MACE, including cardiac death, nonfatal myocardial infarction (MI), or target vessel revascularization (TVR).

### Statistical analysis

Continuous variables are reported as either mean ± SD or median with IQR (25th–75th percentile), based on their distribution. Normality was assessed by drawing Q-Q plots. Paired continuous variables were evaluated using the paired *t*-test for normally distributed data and the Wilcoxon signed-rank test for non-normally distributed data. Unpaired continuous variables were assessed with the unpaired *t*-test if normally distributed and with the Mann-Whitney *U* test for non-normal distributions. Categorical variables were expressed as frequencies and percentages and analyzed using the chi-square test or Fisher exact test, when appropriate. Key outcomes compared included technical success, procedural success, intraprocedural complications, and in-hospital MACE. We calculated relative risks with 95% CIs for binary outcomes and provided *P* values to assess statistical significance. Kaplan-Meier analysis estimated cumulative MACE at 12-month follow-ups. The significance of differences in the MACE endpoint between groups was assessed with the use of the log-rank test. Therefore, HR was also obtained. All tests were two-sided, with *P* < 0.05 indicating statistical significance. Statistical analyses were conducted using SPSS for Windows (version 25.0, IBM) and R R (version 4.4.3, R Core Team, 2024).

## Results

### Baseline characteristics

Among 579 patients, 45 (7.8%) were treated with DCB after IVL. The baseline characteristics of patients treated with IVL are detailed in Table 1. The median age was comparable between DCB and DES groups (72 [65-77] vs 74 [68-80] years; *P* = 0.07). The majority of the patients were male, with no significant differences between groups (*P* = 0.34). Cardiovascular risk factors like hypertension (84.4% vs 69.9%; *P* = 0.06), dyslipidemia (66.7% vs 53.2%; *P* = 0.13), smoking history (37.8% vs 41.9%; *P* = 0.39), and diabetes mellitus (35.6% vs 33.1%; *P* = 0.80) were similar between DEB and DES groups. A positive history of previous PCI (80.0% vs 42.7%; *P* < 0.01) as well as of previous MI (60.0% vs 34.3%, *P* < 0.01) was significantly higher in the DCB group. No difference in clinical presentations or angina severity was found between the 2 groups.

**Table 1:** Baseline Characteristics

	DCB (n = 45)	DES (n = 534)	*P* Value^a^
Age, y	72 [65-77]	74 [68-80]	0.07
Female	9 (20.0)	141 (27.2)	0.34
BMI	25.9 [23.4-28.6]	26.6 [23.8-29.4]	0.66
Hypertension	38 (84.4)	373 (69.9)	0.06
Dyslipidemia	30 (66.7)	284 (53.2)	0.13
Smoking history	17 (37.8)	224 (41.9)	0.39
Diabetes mellitus	16 (35.6)	177 (33.1)	0.80
Premature FH of CAD^b^	14 (31.1)	129 (24.2)	0.38
LVEF	55 [41-60]	55 [41-55]	0.51
Syntax score	19 [11-31]	19 [12-29]	0.68
Chronic kidney disease (eGFR <60 mL/min/1.73 m^2^)	19 (42.2)	154 (28.8)	0.06
GFR (mL/min)	68 [43-87]	71 [54-85]	0.31
Previous PCI	36 (80.0)	228 (42.7)	**<0.01**
Previous CABG	10 (22.2)	93 (17.4)	0.39
Previous MI	27 (60.0)	183 (34.3)	**<0.01**
Previous stroke/TIA	5 (11.1)	70 (13.1)	0.69
Clinical presentation			
Stable angina	24 (53.3)	253 (47.3)	0.47
Unstable angina	7 (15.6)	56 (10.5)	
NSTEMI	8 (17.8)	132 (24.7)	
STEMI	4 (8.9)	39 (7.3)	
Others	2 (4.4)	53 (9.9)	
Angina pectoris^c^			
Class I	1 (2.2)	20 (3.7)	0.59
Class II	15 (33.3)	184 (34.5)	
Class III	13 (28.9)	124 (23.2)	
Class IV	7 (15.6)	53 (9.9)	
Angina equivalent/unknown	8 (17.8)	79 (14.8)	
Anti-ischemic medication			
Betablockers	28 (62.2)	319 (59.7)	0.74
Nitrates	21 (46.7)	142 (26.6)	<0.01

Values are mean ± SD, median (IQR), or n (%). **Bold** value indicates statistical significant difference.

BMI = body mass index; CABG = coronary artery bypass graft; CAD = coronary artery disease; DCB = drug-coated balloon; DES = drug-eluting stent; eGFR = estimated glomerular filtration rate (using the MDRD [Modification of Diet in Renal Disease] formula); FH = family history; LVEF = left ventricular ejection fraction; MI = myocardial infarction; NSTEMI = non –ST-elevation myocardial infarction; PCI = percutaneous coronary intervention; STEMI = ST-segment elevation myocardial infarction; TIA = transient ischemic attack.

a Family history of CAD before 50 years-old.

b *P* values were calculated for DCB vs DES.

c According to the Canadian Cardiovascular Society grading of angina pectoris.

### Procedural characteristics

Procedural characteristics of patients treated with IVL for both groups are summarized in Table 2. The procedural time and the total contrast volume was comparable between DCB and DES. Radial access was the most common approach in both groups (66.7% vs 77.1%; *P* = 0.33). The left anterior descending artery was the most frequently treated vessel in both groups, although with a trend toward higher prevalence in the DES group (30.5% vs 42.4%; *P* = 0.06). CTOs were significantly more common in the DCB group (17.8% vs 6.8%; *P* < 0.01), as were ISR lesions (60.0% vs 29.6%; *P* < 0.01). Use of rotational atherectomy (6.7% vs 13.2%; *P* = 0.24) and cutting balloons (0% vs 0.9%; *P* = 0.53) before IVL were comparable between the 2 groups. The rate of IVL crossing success was comparable between the DCB and DES (100% vs 98.3%; *P* = 0.45). Post-IVL high-pressure balloon dilatation was significantly less frequent in the DCB group (73.3% vs 92.4%; *P* = 0.03). Intraprocedural complications were low, with no significant difference between the considered groups (8.9% vs 5.8%). We had 1 acute vessel closure, which occurred after rotational atherectomy but before IVL likely due to plaque embolization. Following administration of intracoronary vasodilators and saline, coronary flow improved, and the final TIMI flow was grade 2 at the end of the procedure. Thus no bailout stenting was required. Regarding the case of severe dissection, this occurred in the context of a CTO and was classified as a type D dissection. As part of a planned “investment procedure”, we opted not to implant a stent. The patient was scheduled for angiographic follow-up at 3 months, in line with our institutional protocol, and the vessel was patent.

**Table 2:** Procedural Characteristics

	DCB (n = 45)	DES (n = 564)	*P* Value^a^
Procedural time (min)	73 [52-101]	81 [60-111]	0.10
Contrast volume (mL)	150 [120-230]	170 [130-230]	0.16
Access			
Radial	30 (66.7)	412 (77.1)	0.33
Femoral	15 (33.3)	120 (22.5)	0.07
Brachial	0 (0)	2 (0.4)	0.68
Target lesion	46	608	
Left main	2 (4.3)	64 (10.5)	0.15
Left anterior descending artery	14 (30.5)	258 (42.4)	0.06
Circumflex	9 (19.6)	88 (16.5)	0.44
Right coronary artery	21 (45.6)	191 (35.8)	0.08
Arterial graft	0 (0)	1 (0.2)	0.78
Venous graft	0 (0)	6 (1.3)	0.49
Bifurcation	6 (13.3)	129 (24.1)	0.14
CTO	8 (17.8)	36 (6.8)	**<0.01**
In-stent	27 (60.0)	158 (29.6)	**<0.01**
Ostial lesions	7 (15.6)	138 (24.8)	0.18
Inotropes	0 (0)	19 (35.6)	0.20
Vasopressor	3 (6.7)	12 (2.2)	0.08
Need for mechanical support	2 (4.4)	14 (2.6)	
IABP	0 (0)	2 (0.4)	0.48
Impella	2 (4.4)	9 (1.7)	
VA-ECMO	0 (0)	4 (0.7)	
Rotational atherectomy (before IVL)	3 (6.7)	71 (13.2)	0.24
Cutting balloon (before IVL)	0 (0)	5 (0.9)	0.53
Pre-IVL largest balloon (mm)	3.0 [3.0-3.5]	3.0 [2.5-3.5]	0.44
Pre-IVL high-pressure dilatation	39 (86.7)	510 (95.5)	0.12
Pre-IVL maximum pressure dilatation (atm)	3.5 [3.0-4.0]	3.5 [3.0-4.0]	0.69
IVL crossing success	45 (100)	525 (98.3)	0.45
Post-IVL high-pressure dilatation	33 (73.3)	499 (92.4)	**0.03**
Post-IVL largest balloon (mm)	3.5 [3.0-4.0]	3.5 [3.5-4.0]	0.11
Post-IVL maximum pressure dilatation (atm)	20 [16-24]	20 [18-22]	0.43
Total stent length (mm)	-	38 [29-59]	-
Stent maximum diameter (mm)	-	3.5 [3.5-4.0]	-
Bailout DES	0 (0)	-	-
DEB type (drug delivered)	-	-	-
Paclitaxel-coated	31 (68.9)		
Sirolimus-coated	14 (31.1)		
Intraprocedural complications	4 (8.9)	31 (5.8)	0.41
Severe dissections (D - E − F)	1 (2.2)	8 (1.5)	0.71
Abrupt vessel closure	1 (2.2)	5 (0.9)	0.51
Perforation	0 (0)	8 (1.5)	0.41
Hemodynamic instability (intervention)	2 (4.5)	7 (1.3)	0.11
Complication IVL-related	0 (0)	7 (1.3)	0.29

Values are mean ± SD or median (IQR). **Bold** value indicates statistical significant difference.

CTO = chronic total occlusion; IABP = intra-aortic balloon pump; IVL = intravascular lithotripsy; VA-ECMO = veno-arterial extracorporeal membrane oxygenation; other abbreviations as in Table 1.

a *P* values were calculated for DCB vs DES.

### Imaging characteristics

The intracoronary imaging characteristics for patients treated with IVL are detailed in Table 3. Intracoronary imaging was used in a comparable proportion of patients in the DCB and DES groups (53.3% vs 50.6%; *P* = 0.41). The choice of imaging modality was also similar, with IVUS being the most frequently used device in both groups (44.4% vs 47.4%, *P* = 0.82), whereas OCT was used more frequently in the DCB group, although the difference did not reach statistical significance (8.9% vs 3.2%; *P* = 0.12). Pre-IVL area stenosis (73% [65%-76%] vs 71% [60%-79%]; *P* = 0.81) and the maximum persistent calcium angle (360° [282-360] vs 360° [234-360]; *P* = 0.13) were comparable. Postprocedure, the MSA/MLA was slightly lower in the DCB group but did not reach statistical significance (8.67 [6.90-11.78] mm^2^ vs 9.68 [7.70-11.40] mm^2^; *P* = 0.36). QCA characteristics are detailed in Supplemental Table 1.

**Table 3:** Intracoronary Imaging Characteristics

	DCB (n = 24)	DES (n = 270)	*P* Value^a^
Intracoronary imaging used	24/45 (53.3)	270/534 (50.6)	0.41
Intracoronary imaging devices			
IVUS	20 (44.4)	253 (47.4)	0.82
OCT	4 (8.9)	17 (3.2)	0.12
Reference vessel diameter (mm)	4.1 [3.9-4.5]	4.1 [3.7-4.5]	0.63
Pre-IVL minimum lumen diameter (mm)	2.0 [1.8-2.5]	1.9 [1.7-2.3]	0.28
Pre-IVL minimum lumen area (mm^2^)	3.62 [2.93-5.10]	3.93 [2.80-4.90]	0.97
Pre-IVL diameter stenosis (%)	50 [44-53]	51 [44-59]	0.43
Pre-IVL area stenosis (%)	73 [65-76]	71 [60-79]	0.81
Max persistent Ca^2+^ angle (^o^)	360 [282-360]	360 [234-360]	0.13
Post-minimum stent/lumen area (mm^2^)	8.67 [6.90-11.78]	9.68 [7.70-11.40]	0.36
Post-stent expansion at MSA (%)	-	74 [64-82]	-
Post-eccentricity index at MSA	-	0.11 ± 0.02	-
Persistent Ca^2+^ fracture	64%	42%	**0.02**

Values are mean ± SD, median (IQR), or n (%). **Bold** value indicates statistical significant difference.

IVUS = intravascular ultrasound; MSA = minimum stent area; OCT = optical coherence tomography; other abbreviations as in Tables 1 and 2.

a *P* values were calculated for DCB vs DES.

### Procedural and clinical outcomes

Table 4 shows procedural and clinical outcomes. The median follow-up was 365 (225-365) days. Lost at 30 days follow-up was 2 (4.4%) and 21 (3.9%) for DCB and DES, respectively. Procedural success (84.4% vs 89.8%; *P* = 0.98) were comparable between DCB and DES. At the in-hospital phase, no MACE events were recorded in the DCB group, whereas the DES group had a low rate of MACE (0% vs 2.1% *P* = 0.33), driven by cardiac death (0% vs 1.7%) and TVR (0% vs 0.4%), with no occurrences of MI. At 30 days, MACE rates remained low and similar between groups (2.2% vs 3.2%; *P* = 0.99). Cardiac death was rare (0% vs 1.9%; *P* = 0.74). TVR occurred at comparable rates (2.2% vs 1.3%; *P* = 0.48). At 12 months, MACE rates (11.1% vs 7.5%; *P* = 0.38), cardiac death (2.2% vs 2.4%; *P* = 0.99), MI (2.2% vs 1.9%; *P* = 0.96), and TVR (8.9% vs 4.5%; *P* = 0.26) were also comparable between DCB and DES.

**Table 4:** Technical and Clinical Outcomes

	DCB (n = 45)	DES (n = 534)	RR (95% CI)^a^	*P* Value^b^
Procedural success	38 (89.4)	469 (89.8)	0.96 (0.84-1.09)	0.98
In-hospital				
MACE	0 (0)	11 (2.1)	-	0.33
Cardiac death	0 (0)	9 (1.7)	-	0.80
MI	0 (0)	0 (0)	-	-
TVR	0 (0)	2 (0.4)	-	0.68
30-days				
MACE	1 (2.2)	17 (3.2)	0.70 (0.01-5.13)	0.96
Cardiac death	0 (0)	10 (1.9)	-	0.74
MI	1 (2.2)	4 (0.7)	2.97 (0.34-25.99)	0.33
TVR	1 (2.2)	7 (1.3)	1.70 (0.21-13.48)	0.48
6-months				
MACE	2 (4.4)	32 (6.0)	0.74 (0.18-2.99)	0.92
Cardiac death	1 (2.2)	12 (2.2)	0.99 (0.13-7.43)	1.00
MI	1 (2.2)	7 (1.3)	1.70 (0.21-13.48)	0.48
TVR	1 (2.2)	19 (3.6)	0.62 (0.01-4.56)	0.96
12-months				
MACE	5 (11.1)	40 (7.5)	1.48 (0.62-3.57)	0.38
Cardiac death	1 (2.2)	13 (2.4)	0.91 (0.12-6.82)	0.99
MI	1 (2.2)	10 (1.9)	1.19 (0.16-9.06)	0.96
TVR	4 (8.9)	24 (4.5)	1.98 (0.72-5.45)	0.26

Values are n (%).

MACE = major adverse cardiac events; RR = relative risk; TVR = target vessel revascularization; other abbreviations as in Table 1.

a RR and CI for ≥80 vs < 80 years.

b *P* values were calculated for DCB vs the DES; *P* values were calculated for the elderly vs the nonelderly population.

## Discussion

**Figure 1:**
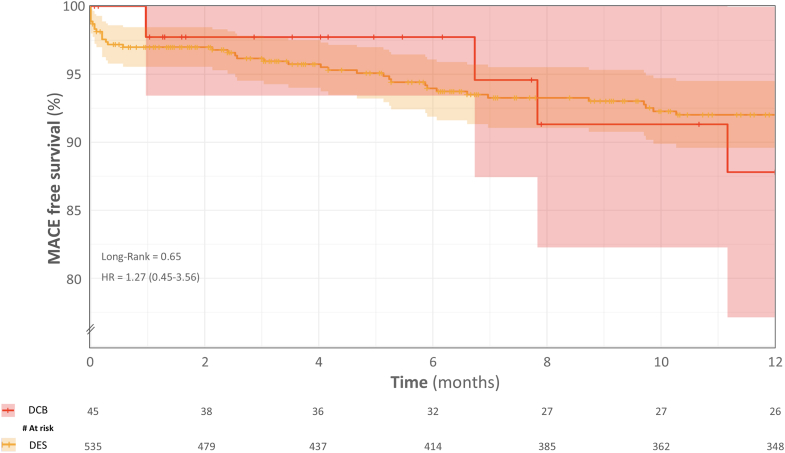
**Kaplan-Meier Curves Showing 1-Year Major Adverse Cardiac Events Outcomes Between Drug-Coated Balloon and Drug-Eluting Stent Strategy After Intravascular Lithotripsy** The curves indicate no significant differences in event-free survival between the 2 groups. DCB = drug-coated balloon; DES = drug-eluting stent; MACE = major adverse cardiac events.

The aim of this study was to provide a real-world comparison between DCB and DES after IVL for the treatment of calcified coronary lesions. Key findings are as follows: 1) technical and procedural success rates were high and comparable between the DES and DCB groups; 2) no significant difference in MACE was observed at the 1-year follow-up (Figure 1); 3) no bailout DES implantation was required in the DCB arm; and 4) CTO and ISR were significantly more often treated with DEB (Central Illustration).

**Figure 2:**
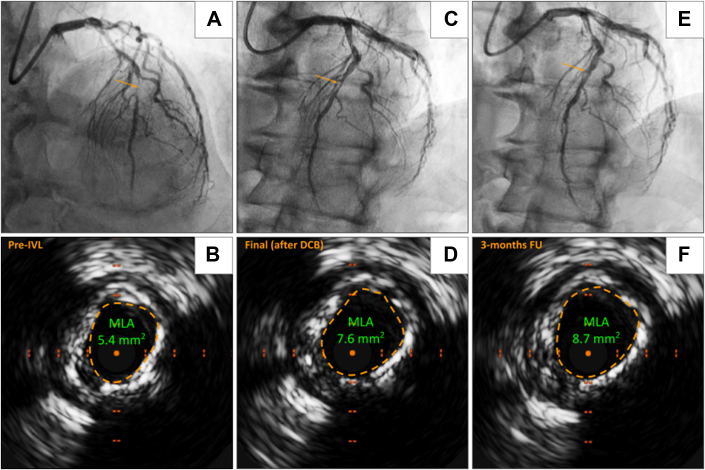
**Chronic Total Occlusion of the LAD Treated With Intravascular Lithotripsy and Drug-Coated Balloon** (A) The pre-IVL coronary angiogram shows a calcified occlusion of the mid LAD with retrograde flow from the CX. (B) After successful anterograde wiring and balloon predilatation, IVUS was performed, revealing severe, long calcifications. (C, D) A 1:1 IVL was used, and the lesion was treated with a long DCB, achieving satisfactory acute gain and MLA. (E) At 3-month follow-up, vessel remodeling occurred. (F) IVUS confirmed the results and the increased luminal gain. IVL = intravascular lithotripsy; LAD = left anterior descending artery; MLA = minimum lumen area; other abbreviations as in Figure 1.

**Central Illustration:**
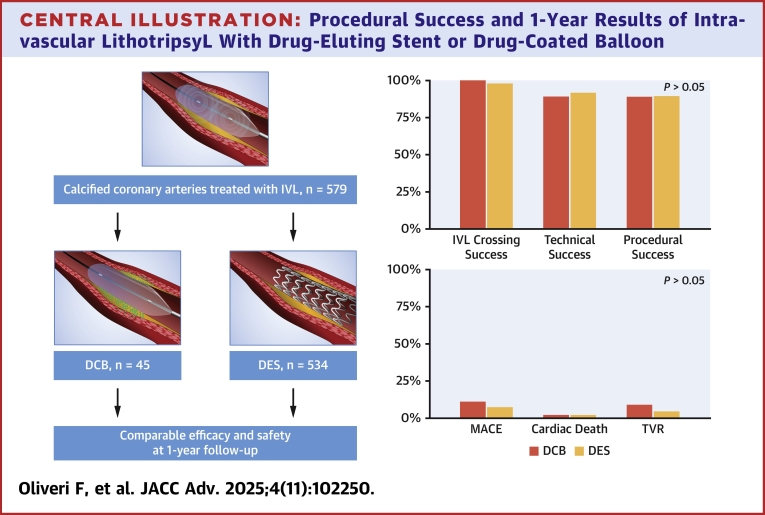
**Procedural Success and 1-Year Results of Intravascular LithotripsyL With Drug-Eluting Stent or Drug-Coated Balloon** DCB = drug-coated balloon; DES = drug-eluting stent; IVL = intravascular lithotripsy; MACE = major adverse cardiac events; TVR = target vessel revascularization.

Coronary artery calcification is a highly prevalent condition that poses significant procedural challenges during PCI. First, calcium reduces vessel compliance, which can lead to suboptimal PCI results, including an increased risk of stent underexpansion and malapposition. These factors, in turn, heighten the risk of ISR and stent thrombosis, ultimately compromising long-term outcomes.^17^, ^18^, ^19^, ^20^ Second, arterial calcium, primarily composed of hydrophilic hydroxyapatite, interacts poorly with the lipophilic drugs in DES and DCB, thereby impairing pharmacokinetics and reducing efficacy.^7^ Given these challenges, DES have historically been the preferred strategy in calcified coronary lesions due to their radial force, which helps counteract the tendency of calcified lesions to recoil. However, several limitations must be considered. First, not all coronary artery calcification patterns present the same prevalence, technical challenges or clinical impact.^21^ For example, in calcium nodules, their eccentric and irregular morphology may increase stent underexpansion or malapposition while increasing the risk of perforations, since the balloon dilation works mainly through the eccentric expansion of the healthy vessel wall opposite the calcified wall.^21^ Second, stents, although providing immediate luminal gain, may interfere with the vessel's natural remodeling capacity. Vessel remodeling, referring to the dynamic changes in the coronary artery diameter and structure over time after an intervention, plays a crucial role in long-term outcomes.^22^ The permanent rigid metallic scaffold may limit adaptive changes, potentially leading to late stent malapposition, chronic inflammation or neointimal hyperplasia. To address these limitations, DCBs have emerged as a promising alternative, offering the potential to combine the stent-free advantages of POBA with the proven efficacy of DES.^3^, ^4^, ^5^ However, their role in treating calcified coronary lesions remains less established. One advantage of DCBs in calcified lesions is their greater flexibility for vessel remodeling, allowing the artery to adapt over time without being constrained by a rigid stent, particularly in cases where positive remodeling is expected. This is especially important in the modern treatment of CTO, where subintimal tracking and re-entry associated with investment procedures are showing promising outcomes.^23^^,^^24^ This approach aligns with the dynamic nature of CTO lesions, where vessel remodeling occurs following recanalization.^25^ In line with this concept, our study observed a higher prevalence of CTOs treated with DCB than DES, reflecting evolving trends in CTO management (Figure 2). Beyond its established role in high-bleeding-risk patients, DCB may also offer advantages in complex anatomical settings, including calcified bifurcation and ostial lesions.^6^^,^^26^ A DCB-based strategy in bifurcation PCI may help preserve the bifurcation anatomy, reduce procedural complexity and duration, and be particularly beneficial in a hybrid approach, where DES is used for the main branch and DCB for the side branch. On the other hand, in ostial lesions, DCB may avoid the rewiring challenges and lower the risk of thrombosis associated with protruding DES struts beyond the ostium.

One key limitation is the lack of radial force, which makes DCBs more susceptible to elastic recoil, particularly in highly calcified lesions. However, these challenges can be mitigated with calcium modification techniques, which enhance DCB delivery, facilitate vessel expansion, and improve drug absorption in calcified lesions. In balloon-uncrossable lesions, atherectomy has demonstrated efficacy in facilitating device delivery. Recently, some studies have suggested that a DCB-based strategy following rotational atherectomy yields procedural and clinical outcomes comparable to DES, although the sample size is underpowered to draw definitive conclusions.^27^^,^^28^ For balloon-crossable lesions, DCB may be a viable option following IVL. Our study supports this hypothesis, demonstrating favorable outcomes with DCB after IVL. The multiple macrofractures and microfractures induced by IVL provide 2 key advantages: 1) improved vessel compliance, reducing the need for high radial force to maintain luminal expansion; and 2) enhanced drug diffusion, allowing for more effective drug penetration into the vessel wall, thereby promoting its therapeutic efficacy. The minimal procedural complications confirm the safety of DCB implantation after IVL, opening to a modern approach for the treatment of calcified coronary lesions.

## Study Limitations

The present study has several limitations. First, although the study had a prospective observational design, it did not eliminate the risks of allocation bias or lack of blinding. Second, despite the well-established role of intracoronary imaging in guiding complex PCI, its use in our study was suboptimal, which may have affected lesion assessment and treatment success. Third, the relatively limited sample size for clinical outcomes reduces the statistical power of our findings and their generalizability to broader clinical practice.

## Conclusions

In calcified coronary lesions treated with IVL, DCB demonstrated comparable procedural success to DES. The low rate of procedural complications and long-term MACE reinforces the safety of the “crack and drug” strategy, supporting its potential role as a modern approach for the treatment of calcified coronary lesions.

## Funding support and author disclosures

This work was funded through a research grant from Shockwave Medical. Funders were not involved in any aspect of the study, including its design, collection, analysis and interpretation of the data, or the writing of the manuscript. The 10.13039/501100005038Department of Cardiology of the Leiden University Medical Center received unrestricted research grants from 10.13039/100011949Abbott Vascular, 10.13039/100004326Bayer, 10.13039/501100005035Biotronik, 10.13039/100008497Boston Scientific, 10.13039/100006520Edwards Lifesciences, 10.13039/100006775GE Healthcare, and 10.13039/100004374Medtronic. Dr Paradies received unrestricted research grants from 10.13039/100011949Abbott Vascular, 10.13039/100014833SMT and Terumo via the Institution and consultancy fee from 10.13039/100011949Abbott Vascular, 10.13039/501100004191Novo Nordisk, 10.13039/100014833SMT, Elixir Medical, and 10.13039/100008497Boston Scientific. Dr Claessen received consultancy fees from 10.13039/100020297Abiomed, 10.13039/100011949Abbott Vascular, 10.13039/100002429Amgen, 10.13039/100007535BBraun, 10.13039/100016242Boston Scientific, Philips and Sanofi and received research funding from Philips, 10.13039/501100004191Novo Nordisk, 10.13039/100007535BBraun and 10.13039/100018503Infraredx. Dr van der Kley received consultancy fees from 10.13039/100006520Edwards Lifesciences and 10.13039/100011949Abbott Vascular. Dr Jukema/his department has received research grants from and/or was speaker (with or without lecture fees) on a.o.(CME accredited) meetings sponsored/supported by 10.13039/100000046Abbott, Amarin, 10.13039/100002429Amgen, Athera, 10.13039/501100005035Biotronik, 10.13039/100016242Boston Scientific, Dalcor, 10.13039/501100022274Daiichi Sankyo, 10.13039/100006520Edwards Lifesciences, GE Healthcare Johnson and Johnson, 10.13039/100004312Lilly, 10.13039/100004374Medtronic, Merck-Schering-Plough, 10.13039/100004336Novartis, 10.13039/501100004191Novo Nordisk, 10.13039/100004319Pfizer, 10.13039/100004337Roche, Sanofi Aventis, Shockwave Medical, the Netherlands Heart Foundation, CardioVascular Research the Netherlands (CVON), the Netherlands Heart Institute, and the European Community Framework KP7 Programme. Dr Montero-Cabezas received a research grant from Shockwave Medical and speaker fees from 10.13039/100020297Abiomed, 10.13039/100008497Boston Scientific, and 10.13039/100020501Penumbra Inc. All other authors have reported that they have no relationships relevant to the contents of this paper to disclose.
