# Supplementary material for: Drug-Coated Balloon After Intravascular Lithotripsy: Procedural and Clinical Outcome of the “Crack and Drug” Strategy
Source: JACC Adv. 2025 Oct 24;4(11):102250. doi: 10.1016/j.jacadv.2025.102250 (PMC12593658; doi:10.1016/j.jacadv.2025.102250)
Supplement: Supplementary Material [file mmc1.pdf]

Table S1 QCA angiographic characteristics

	**DCB** (n= 37)	**DES** (n= 481)	**p-value‡**
**Reference vessel diameter** (mm)	3.00 [2.65-3.70]	3.20 [2.80-3.70]	0.42
**Pre-PCI minimum lumen diameter** (mm)	0.83 ± 0.13	1.04 ± 0.03	0.10
**Pre-PCI minimum Lumen area** (mm^2)	1.08 ± 0.32	1.22 ± 0.08	0.62
**Pre PCI diameter stenosis** (%)	76 [55-98]	70 [55-83]	0.18
**Pre PCI area stenosis** (%)	94 [80-99]	91 [79-97]	0.15
**Post PCI Minimum lumen area** (mm^2)	5.73 ± 0.55	6.94 ± 0.14	**0.02**
**Acute gain** (mm)	1.71 ± 0.17	1.89 ± 0.04	0.25

Values are mean ± SD or median (IQR); ‡ p-values were calculated for DCB vs the DES.
